# The variability of competitive performance and pacing strategies in different rounds of the 400 m and 800 m freestyle swimming races at the 2017–2024 World Swimming Championships

**DOI:** 10.3389/fspor.2024.1496878

**Published:** 2024-10-30

**Authors:** Junhui Fang, Yunpeng Li, Yan Cheng

**Affiliations:** ^1^ Department of Sport Training, China Institute of Sport Science, Beijing, China; ^2^ School of Physical Education, Guizhou Normal University, Guiyang, China

**Keywords:** swimmers, freestyle race, competitive performance, pacing strategies, heats and finals

## Abstract

**Introduction:**

This study aims to analyze the competitive performance and pacing strategies (PS) of medalists and non-medalists in different rounds of the 400 m and 800 m freestyle at the World Swimming Championships.

**Method:**

The 2017–2024 World Swimming Championships and 161 elite swimmers were selected. First, intra-athlete coefficients of variability (CVs) were evaluated using linear mixed effects modeling and changes in competitive performance (Δ); second, descriptive statistics of position lap time; finally, a computer algorithm was used to obtain PS, then a two-way ANOVA was performed.

**Result:**

(i) The PS was effective in 87.5% of the swimmers in the finals compared to the heats (CVs > 0.5%), but 73.8% of the males and 86.8% of the females showed an improvement in performance prior to the finals (Δ < 0); (ii) Gold medalists had an average position no lower than the top 2 and established themselves in the top 3 positions more than 90% of the time, aiming to remain in the top 3 until the final 100 m if they were to win a medal; (iii) The female swimmers in 400 m were more in the heats utilize the inverted-J (race velocity change curve profile as inverted-J), men for the fast-start-even, in the final, female remain the inverted-J, men change to the U-shaped (race velocity change curve profile as U-shaped), and in the 800 m, the swimmers were unified adopt the U-shaped.

**Discussion:**

The elite swimmers who qualified for the finals performed better in the heats and semifinals because their PS were more effective. Others, however, did not have a chance to reach the finals because their PS efficiency was lower, and their competitive performance improved less or even regressed.

## 1 Introduction

Swimming is one of the few sports in the Olympic Games that involves repetitive movements over a limited distance in the same event, running back and forth, requiring competitors to qualify for advancement from heats or semifinals to reach the finals. There is evidence that swimmers perform more consistently in the same event (1), but improving the variability in performance between rounds to drive the best performances to the finals would greatly increase the chances of winning a medal (2). However, it is inevitable that fatigue will occur in any human activity, particularly in high-intensity competition, there is a noticeable decrease in the speed of swimmers as the race distance increases (3). In order to perform at their optimal level, swimmers must employ an effective pacing strategy (4). The term “pacing strategy” (PS) is defined as the rate at which the body regulates and distributed effectively metabolic energy while limiting premature fatigue during exercise (5), allowing athletes to perform at their optimal level in the appropriate scenario (6), as it is a critical element in determining success or failure (7, 8). Whether in the pool, open water (9), or triathlon (10), Abbiss & Laursen (2008) (7) previously described and defined six different PS, these strategies are prevalent in chronological sports, with the specific approach varying depending on the movement, event or distance.

When examining the strategy used by swimmers in long-distance swimming events, such as the 1,500 m freestyle, it is evident that the U-shaped strategy is often used, suggesting that they start and finish the race faster and with more intensity, but slowdown in the middle laps (11). In contrast, for shorter distances, such as the 200 m freestyle distance, swimmers typically adopt a fast-start-even strategy, accelerating at the start and then speeding up their stroke rate (SR), maintaining as small a sustained decrease in speed as possible on each lap (12). This approach is observed in both medal and non-medal swimmers. In addition, short-distance 50 m and 100 m freestyle swimmers use an all-out strategy to prevent a decrease in speed (5). Variability in middle-distance freestyle is relatively less fixed, e.g., a greater proportion of swimmers in 400 m and 800 m races than in long-distance races improve their competitive performance by altering their PS (13), this strategy is undoubtedly much less variable than in shorter-distance races. In other words, middle-distance swimmers either adopt a U-shaped strategy or other curvilinear types of strategies. This depends on an individual's type of training performed, the training phase, and the swimmer's psychological resilience and physiological conditions (2). It also indicates that the PS employed in a mid-distance freestyle race has a significant impact on the final sprint and is a critical determinant of success.

It is observed that the role of swimming PS is often emphasized in the final round. Indeed, as described in the definition of PS in this study, its influence can be significant in the rounds leading up to the finals. Although recent studies have analyzed results in the 200 m (14), 400 m (15, 16), 800 m (11), and 1,500 m (17, 18) finals, only a few studies (19) have examined in depth the changes in competitive performance and PS between different rounds of the 100 m and 200 m races, i.e., analyzing heats and semifinals. There have been calls for the use of high-level competition data to study PS outside of real-world and laboratory conditions (20, 21). However, researchers have found that in a study of different rounds of the 400 m (22), only 13 individuals from one race were included in the data analysis, and in a study of the 800 m freestyle (23), the participants were non-elite swimmers. Both were cross-sectional datasets.

It is therefore necessary to examine the most recent consecutive years to differentiate between long and short distances and to examine the differences in competitive performance between different rounds. It is also possible to examine the differences in PS choices between medalists and non-medalists. Finally, this key information can be used by coaches and swimmers in these events during heats and finals as a reference for training strategies and race plans. This is particularly relevant for the preparation of major international events and will contribute to the theoretical basis for the development of scientific researchers in other sports. As in the previous study (24), which indicated that the choice of a fast-start-even and a parabolic strategy for the 400 m freestyle final can be effective in achieving times close to the world record (WR), coaches and swimmers were advised to use a combination of these two strategies during pre-competition training. Therefore, the purpose of this study was to analyze the PS and competitive performance of medalists and non-medalists in different rounds of the 400 m and 800 m freestyle at the World Swimming Championships. This involved: (1) analyzing the coefficients of variability (CVs) and the effective change in competitive performance (Δ) between swimmers in different rounds; (2) analyzing the rounds position of swimmers; and (3) analyzing the importance of PS and its relationship with competitive performance.

## 2 Materials and methods

### 2.1 Participant

A total of 161 (80 males and 81 females/30 medalists and 131 non-medalists) elite swimmers from 27 countries were selected according to the official rankings retrieved from the website www.worldaquatics.com (as of May 1, 2024) in the 400 m and 800 m male and female freestyle from five World Swimming Championships. The medalists refer to athletes who have won gold, silver and bronze medals in the finals, while non-medalists refer to athletes who have not won the aforementioned medals. Of these, 40 males [age: 22.7 ± 2.3 years] [mean ± standard deviation (SD)] and 40 females [age: 20.8 ± 3.7 years] participated in the 400 m freestyle; 40 males [age: 23.2 ± 2.7 years]) and 41 females [age: 21.8 ± 3.1] participated in the 800 m freestyle. The mean age of all swimmers was 22.1 ± 3.1 years. All results were converted to seconds. This study included heats and finals results from the 2017 World Championships in Budapest (Hungary), the 2019 World Championships in Gwangju (Korea), the 2022 World Championships in Budapest (Hungary), the 2023 World Championships in Fukuoka (Japan), and the 2024 World Championships in Doha (Doha). A total of 13 records have been broken in the World Championships, included 3 Championship Records (CR), 2 European Record (ER), 3 Oceanian Record (OC), 1 African Record (AF), 1 Asian Record (AS), 2 Americas Record (AM) and 1 World Record (WR). The WR data for the female and male 400 m and 800 m freestyle used for comparison is sourced from the Olympic Games Paris 2024 swimming results report by World Aquatics (pp.19–20).

### 2.2 Data collection

For each race, two rounds of split time results were collected to analyze competitive performance and changes in the PS from one round to the next. Two researchers independently recorded the swimmers’ data, including “name”, “gender”, “age”, “distance”, “lap”, “ranking”, “split time”, and “race time”. The kappa coefficient of 1 (*P* < 0.001) indicates that the extracted data were identical between the researchers.

### 2.3 Data analysis

The variability in athlete performance between events is also referred to as intra-athlete coefficients of variation (CVs), according to Stewart & Hopkins (2000) (1), a 0.5% change in CVs means that the athlete's PS is valid. In this study, two different CVs were calculated: (1) heats to finals (H-F) and (2) the split results of heats to finals [e.g., H-F (Split 1)], using the following formulas:

(1) $$CV_{H–F}=\frac{\mathrm{Standard}\;\mathrm{deviation}\;(H–F)}{\mathrm{Mean}\;(H–F)}\;*\;100$$

(2) $$CV_{\mathrm{Splits}}=\frac{\mathrm{Standard}\;\mathrm{deviation}\;[e.g.,\;\;H–F(\mathrm{Split}\;1)]}{\mathrm{Mean}\;[e.g.,\;H–F(\mathrm{Split}\;1)]}\;*\;100$$

The relative change in competitive performance (Δ) determines a swimmer's strategic control during two consecutive rounds of high-intensity competition, and when Δ is an increase, no change, or a decrease, the corresponding results are less than, equal to, or greater than 0, respectively (25), with the following formulas:

(3) $$\Delta_{H-F}=\frac{\mathrm{Round}\;2\;\mathrm{performance}\text{-}\mathrm{Round}\;1\;\mathrm{performance}}{\mathrm{Round}\;1\;\mathrm{performance}}$$

**Figure 1:**
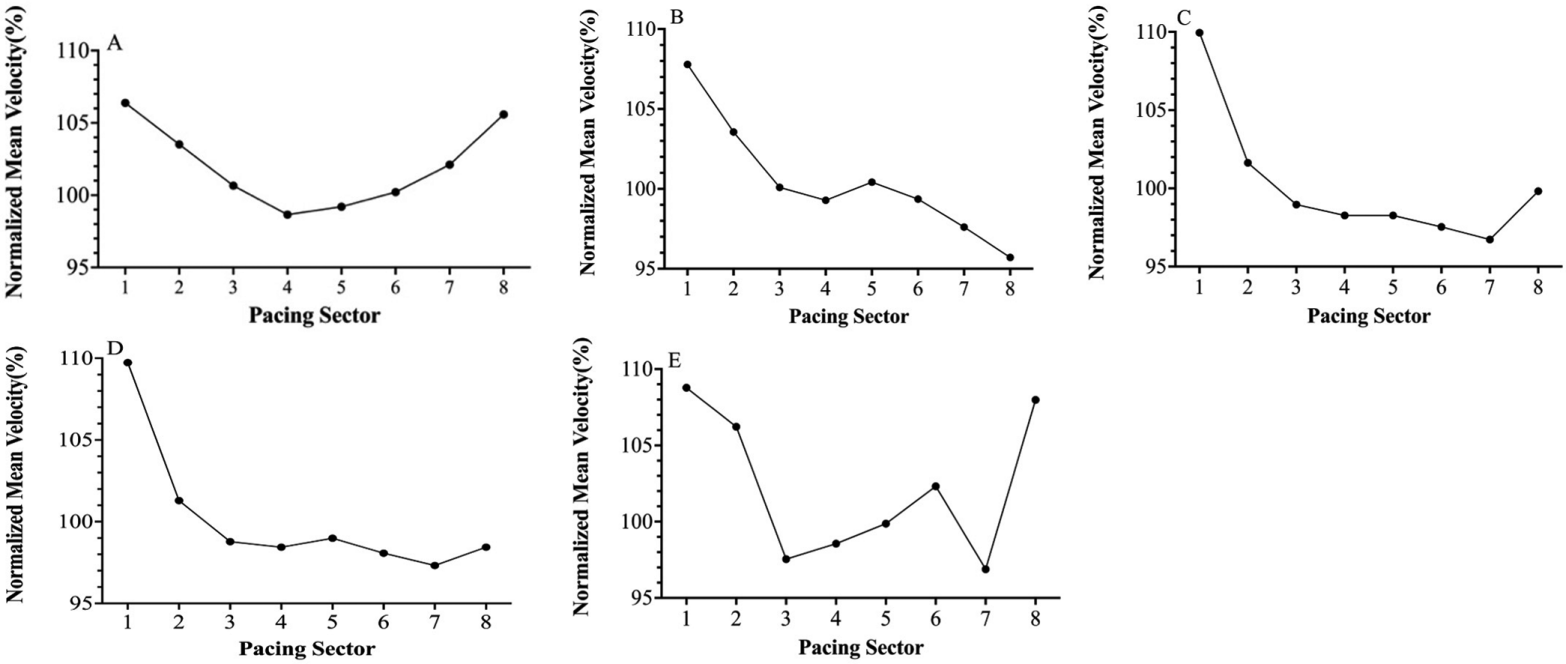
**(A–E)** Pacing strategies of competitive swimming (U-shaped **(A)**, positive **(B)**, (inverted-J) **(C)**, fast-start-even **(D)**, Variable **(E)**).

The PS classification for each race profile was determined using an algorithm from OpenOffice 3.2.1 Calc (Oracle Corp., Redwood Shores, Redwood City, CA). Although the validity of the algorithm needs to be verified, it represents an objective approach to classifying PS. The representation of PS is partly derived from research based on Abbiss & Laursen (2008) (7), as shown in Figure 1, and partly from actual competitions where swimmers combine other pacing in different forms, such as “positive + sprinting” (Figure 1C), to form an inverted-J strategy, only four (A–D) of which were frequently used by elite swimmers. Each strategy is modeled by normalizing the speed, i.e., the percentage obtained by comparing the split speed to the average speed (26). For example, if the average race speed is 1.8 m/s, then for a particular pacing sector (e.g., Lap 1 is 1.98 m/s), the normalized treatment of 1.98 m/s is 109.95% (1.98/1.8). The algorithm automatically classifies PS when it recognizes pacing profiles at different distances for certain values, and this approach is widely accepted. The modeling operation of these key PSs is the same as in previous studies (27).

### 2.4 Statistical procedures

In order to assess whether the variability of competitive performance between different rounds is related to the level of the swimmer, the split times of all swimmers were entered into equations (1), (2) and (3) and evaluated by applying a linear mixed effects model, the same methodology used in previous studies (1, 2, 19). The pacing strategy classification is analyzed using a machine algorithm and then subjected to descriptive statistics. International swimming races were classified by gender (male and female), distance (400 m and 800 m), group (medalists and non-medalists) and round (heats and finals). All races were counted according to eight laps, i.e., eight 50 m for 400 m and eight 100 m for 800 m, it is classically possible to analyze each lap (28), and the analyzed data were expressed as mean ± standard deviation (M ± SD) and 95% confidence intervals (95% CI), the magnitude of within-group differences (heats to finals) was expressed as effect size (ES), they were calculated by dividing the mean difference (between heats and finals) by the average of their standard deviations for each group. Next, the position of the medal and non-medal swimmers was determined for each lap. Finally, the normality of the complete data distribution was assessed using Shapiro-Wilk, and the data were analyzed for mean squared error using Levene's test. When the result of Levene's test appeared to be *P* > 0.05, a two-way ANOVA (factor: group [medalists and non-medalists] × PS [U-shaped, inverted-J, fast-start-even, positive]) could be performed, which was used to calculate the strategy difference between medalists and non-medalists swimmers in rounds, and the analysis scheme included main effects and interaction effects analyses. *P* < 0.05 was considered statistically significant. Analyses were performed using SPSS 27.0 (IBM, Chicago, IL, USA), and figures were generated using Prims 10 (GraphPad Software, Boston, MA, USA).

## 3 Results

### 3.1 Changes in competitive performance across rounds

The results of the CVs and Δ of the swimmers for different rounds and distances are shown in Table 1. This result showed that the CVs and Δ of the swimmers who reached the finals interacted with each other（*P* < 0.05）, and that 87.5% of the female swimmers in the 400 m event and male swimmers in the 800 m event had CVs greater than 0.5% compared to the heats, but 30% of the male swimmers in the 400 m event. The ES were greater for female swimmers in the 400 m event and for male swimmers in the 800 m event, suggesting that the change from heats to finals resulted in a greater improvement. Therefore, 73.8% of the male swimmers and 86.8% of the female swimmers showed an improvement in competitive performance (Δ < 0) after reaching the final, and it is worth noting that the performance regression occurred in non-medalist swimmers.

**Table 1:** Changes in competitive performance between rounds in 400 m and 800 m freestyle swimming.

Event	Gender	Round			All participants			Medalists		Non - medalists	
		Heats (s) (*n* = 80) [95% CI]	Finals (s) (*n* = 80) [95% CI]	ES	CV (%) [95% CI]	Δ (%) [95% CI]	*P*	CV (%) [95% CI]	Δ (%) [95% CI]	CV (%) [95% CI]	Δ (%) [95% CI]
400 m	Males	255.13 ± 0.87	224.23 ± 1.98	−0.63	0.11 ± 0.09	−0.40 ± 0.73	0.011	0.17 ± 0.05	−0.98 ± 0.30	0.08 ± 0.09	−0.05 ± 0.69
		[224.86–225.40]	[223.62–224.84]		[0.08–0.14]	[−0.62∼−0.17]		[0.15–0.20]	[−1.13∼−0.83]	[0.04–0.11]	[−0.32 – 0.22]
	Females	244.41 ± 2.15	243.14 ± 3.44	−0.46	0.15 ± 0.10	−0.52 ± 0.89	0.001	0.21 ± 0.11	−1.16 ± 0.63	0.12 ± 0.08	−0.14 ± 0.80
		[243.75–245.08]	[242.07–244.20]		[0.12–0.18]	[−0.80∼−0.25]		[0.15–0.26]	[−1.48∼−0.84]	[0.09–0.15]	[−0.45∼0.17]
	Mean	234.77 ± 9.84	233.68 ± 9.91	−0.11	0.13 ± 0.10	−0.46 ± 0.81		0.18 ± 0.09	−1.07 ± 0.49	0.09 ± 0.09	−0.10 ± 0.74
		[232.61–236.93]	[231.53–235.84]		[0.11–0.15]	[−0.64∼−0.28]		[0.16–0.22]	[−1.25∼−0.89]	[0.07–0.12]	[−0.30 – 0.11]
800 m	Males	466.33 ± 2.14	464.50 ± 4.72	−0.53	0.14 ± 0.09	−0.39 ± 0.83	0.005	0.20 ± 0.06	−1.11 ± 0.32	0.10 ± 0.08	0.04 ± 0.74
		[465.67–467.00]	[463.04–465.96]		[0.11–0.16]	[−0.65∼−0.14]		[0.17–0.23]	[−1.27∼−0.95]	[0.07–0.13]	[−0.25∼−0.33]
	Females	505.47 ± 5.27	501.63 ± 7.28	−0.61	0.18 ± 0.10	−0.76 ± 0.85	0.000	0.26 ± 0.08	−1.45 ± 0.47	0.13 ± 0.08	−0.36 ± 0.76
		[503.86–507.09]	[499.40–503.86]		[0.14–0.21]	[−1.02∼−0.50]		[0.22–0.30]	[−1.69∼−1.21]	[0.10–0.16]	[−0.66∼−0.07]
	Mean	486.14 ± 20.10	483.30 ± 19.66	−0.14	0.16 ± 0.10	−0.58 ± 0.86		0.23 ± 0.08	−1.28 ± 0.43	0.12 ± 0.08	−0.18 ± 0.77
		[481.77–490.52]	[479.02–487.58]		[0.14–0.18]	[−0.77∼−0.39]		[0.20–0.26]	[−1.43∼−1.13]	[0.09–0.14]	[−0.38∼−0.05]

The average range of CVs improvement in the 400 m from heats to finals was approximately 0.46%, while in the 800 m it was approximately 0.58% (Table 1), which would increase to 1.07% for the 400 m and 1.43% for the 800 m if only medalists were considered. A further analysis of the swimmers’ specific performance in 400 m and 800 m split times in Table 2. There are very small differences between events, but larger differences between genders (male's ES are much smaller than female). For females, the CVs ranged from 0.17 to 0.34% for the 400 m and from 0.16 to 0.26% for the 800 m; for males, the CVs ranged from 0.13 to 0.40% for the 400 m and from 0.14 to 0.35% for the 800 m.

**Table 2:** Changes in competitive performance between split in *n* 400 m and 800 m freestyle swimming.

Event	400 m							800 m						
	Split	Heats (s) (*n* = 80)	Finals (s) (*n* = 80)	ES	Heats to finals			Split	Heats (s) (*n* = 80)	Finals (s) (*n* = 80)	ES	Heats to finals		
	50 m	[95% CI]	[95% CI]		CV (%)	Δ (%)	*P*	100 m	[95% CI]	[95% CI]		CV (%)	Δ (%)	*P*
					[95% CI]	[95% CI]						[95% CI]	[95% CI]	
Males	Lap 1	25.98 ± 0.38	25.92 ± 0.31	−0.16	0.13 ± 0.10	−0.20 ± 0.89	0.148	Lap 1	56.99 ± 2.02	56.45 ± 1.91	−0.28	0.19 ± 0.14	−0.95 ± 0.88	0.000
		[25.86–26.10]	[25.83–26.02]		[0.09–0.16]	[−0.48∼0.08]			[56.37–57.62]	[55.96–57.04]		[0.14–0.23]	[−1.22∼−0.68]	
	Lap 2	28.19 ± 0.30	28.16 ± 0.26	−0.11	0.14 ± 0.10	−0.10 ± 0.99	0.502	Lap 2	59.60 ± 2.05	59.06 ± 2.03	−0.26	0.17 ± 0.11	−0.90 ± 0.72	0.000
		[28.09–28.28]	[28.07–28.24]		[0.11–0.17]	[−0.41∼0.21]			[58.97–60.24]	[58.43–59.69]		[0.14–0.21]	[−1.13∼−0.68]	
	Lap 3	28.54 ± 0.22	28.51 ± 0.28	−0.11	0.13 ± 0.12	−0.09 ± 0.99	0.550	Lap 3	59.81 ± 2.05	59.41 ± 2.03	−0.20	0.16 ± 0.12	−0.67 ± 0.93	0.000
		[28.47–28.61]	[28.43–28.60]		[0.09–0.17]	[−0.40∼0.22]			[59.18–60.45]	[58.78–60.04]		[0.12–0.20]	[−0.96∼−0.38]	
	Lap 4	28.70 ± 0.25	28.69 ± 0.32	−0.05	0.16 ± 0.15	−0.04 ± 1.22	0.801	Lap 4	59.94 ± 2.06	59.70 ± 2.11	−0.12	0.17 ± 0.13	−0.40 ± 1.13	0.028
		[28.63–28.78]	[28.59–28.79]		[0.11–0.20]	[−0.42∼0.33]			[59.31–60.58]	[59.05–60.35]		[0.13–0.21]	[−0.75∼−0.05]	
	Lap 5	28.65 ± 0.23	28.59 ± 0.36	−0.21	0.17 ± 0.17	−0.21 ± 1.33	0.313	Lap 5	59.74 ± 1.91	59.65 ± 2.11	−0.04	0.14 ± 0.12	−0.15 ± 1.04	0.395
		[28.58–28.72]	[28.48–28.70]		[0.12–0.22]	[−0.63∼0.20]			[59.14–60.33]	[59.00–60.31]		[0.10–0.18]	[−0.47∼0.18]	
	Lap 6	28.76 ± 0.24	28.64 ± 0.36	−0.39	0.17 ± 0.16	−0.40 ± 1.26	0.051	Lap 6	59.76 ± 2.00	59.82 ± 2.14	0.03	0.14 ± 0.13	0.09 ± 1.11	0.595
		[28.69–28.83]	[28.53–28.76]		[0.12–0.22]	[−0.79∼−0.01]			[59.14–60.38]	[59.15–60.48]		[0.10–0.18]	[−0.25∼0.44]	
	Lap 7	28.56 ± 0.27	28.35 ± 0.49	−0.53	0.25 ± 0.26	−0.70 ± 1.92	0.025	Lap 7	59.70 ± 2.22	59.78 ± 2.32	0.03	0.17 ± 0.14	0.12 ± 1.26	0.553
		[28.47–28.64]	[28.20–28.51]		[0.17–0.33]	[−1.29∼−0.10]			[59.02–60.39]	[59.06–60.50]		[0.12–0.21]	[−0.27∼0.51]	
	Lap 8	27.75 ± 0.44	27.36 ± 0.67	−0.71	0.40 ± 0.32	−1.40 ± 2.54	0.001	Lap 8	58.20 ± 2.42	57.89 ± 2.54	−0.12	0.35 ± 0.29	−0.50 ± 2.51	0.202
		[27.62–27.89]	[27.16–27.57]		[0.30–0.50]	[−2.18∼−0.61]			[57.45–58.95]	[59.11–58.68]		[0.26–0.44]	[−1.28∼0.28]	
Females	Lap 1	28.31 ± 0.46	28.16 ± 0.54	−0.29	0.18 ± 0.19	−0.50 ± 1.35	0.023	Lap 1	60.66 ± 0.94	59.79 ± 1.07	−0.86	0.26 ± 0.14	−1.43 ± 0.79	0.000
		[28.16–28.45]	[28.20–28.33]		[0.12–0.23]	[−0.92∼−0.08]			[60.37–60.95]	[59.47–60.12]		[0.21–0.30]	[−1.67∼−1.19]	
	Lap 2	30.42 ± 0.45	30.25 ± 0.47	−0.36	0.18 ± 0.15	−0.54 ± 1.22	0.008	Lap 2	63.48 ± 0.76	62.72 ± 0.94	−0.89	0.23 ± 0.13	−1.19 ± 0.83	0.000
		[30.28–30.56]	[30.11–30.40]		[0.14–0.23]	[−0.92∼−0.16]			[63.25–63.71]	[62.44–63.01]		[0.19–0.26]	[−1.45∼−0.94]	
	Lap 3	30.80 ± 0.40	30.65 ± 0.39	−0.38	0.17 ± 0.12	−0.49 ± 1.05	0.005	Lap 3	63.75 ± 0.68	63.16 ± 0.83	−0.78	0.18 ± 0.13	−0.92 ± 0.83	0.000
		[30.67–30.92]	[30.52–30.77]		[0.13–0.20]	[−0.82∼−0.17]			[63.55–63.96]	[62.91–63.42]		[0.14–0.22]	[−1.18∼−0.67]	
	Lap 4	30.95 ± 0.31	30.86 ± 0.46	−0.21	0.17 ± 0.11	−0.27 ± 1.10	0.136	Lap 4	63.95 ± 0.65	63.47 ± 0.88	−0.63	0.19 ± 0.10	−0.76 ± 0.91	0.000
		[30.85–31.04]	[30.72–31.01]		[0.13–0.20]	[−0.61∼−0.08]			[63.75–64.15]	[63.20–63.74]		[0.16–0.22]	[−1.03∼−0.48]	
	Lap 5	31.07 ± 0.27	30.89 ± 0.48	−0.47	0.19 ± 0.15	−0.57 ± 1.21	0.005	Lap 5	63.82 ± 0.70	63.58 ± 0.99	−0.29	0.16 ± 0.11	−0.38 ± 1.03	0.022
		[30.99–31.15]	[30.74–31.04]		[0.14–0.23]	[−0.95∼−0.19]			[63.61–64.04]	[63.28–63.88]		[0.12–0.19]	[−0.70∼−0.07]	
	Lap 6	31.24 ± 0.28	31.06 ± 0.57	−0.43	0.23 ± 0.19	−0.58 ± 1.58	0.025	Lap 6	63.89 ± 0.74	63.63 ± 1.08	−0.29	0.17 ± 0.12	−0.41 ± 1.12	0.024
		[31.16–31.33]	[30.88–31.24]		[0.17–0.29]	[−1.07∼−0.09]			[63.66–64.12]	[63.30–63.96]		[0.13–0.21]	[−1.45∼−0.94]	
	Lap 7	31.26 ± 0.35	31.02 ± 0.70	−0.47	0.28 ± 0.26	−0.79 ± 2.04	0.020	Lap 7	63.83 ± 0.89	63.66 ± 1.15	−0.17	0.17 ± 0.12	−0.27 ± 1.17	0.152
		[31.15–31.37]	[30.80–31.23]		[0.20–0.36]	[−1.42∼−0.15]			[63.56–64.10]	[63.31–64.01]		[0.13–0.21]	[−0.62∼0.09]	
	Lap 8	30.37 ± 0.50	30.24 ± 0.66	−0.22	0.34 ± 0.29	−0.40 ± 2.53	0.301	Lap 8	62.08 ± 1.22	61.62 ± 1.29	−0.37	0.34 ± 0.25	−0.72 ± 2.28	0.043
		[30.21–30.52]	[30.03–30.44]		[0.25–0.43]	[−1.18∼0.39]			[61.71–62.45]	[61.22–62.01]		[0.27–0.42]	[−1.42∼−0.03]	

**Figure 2:**
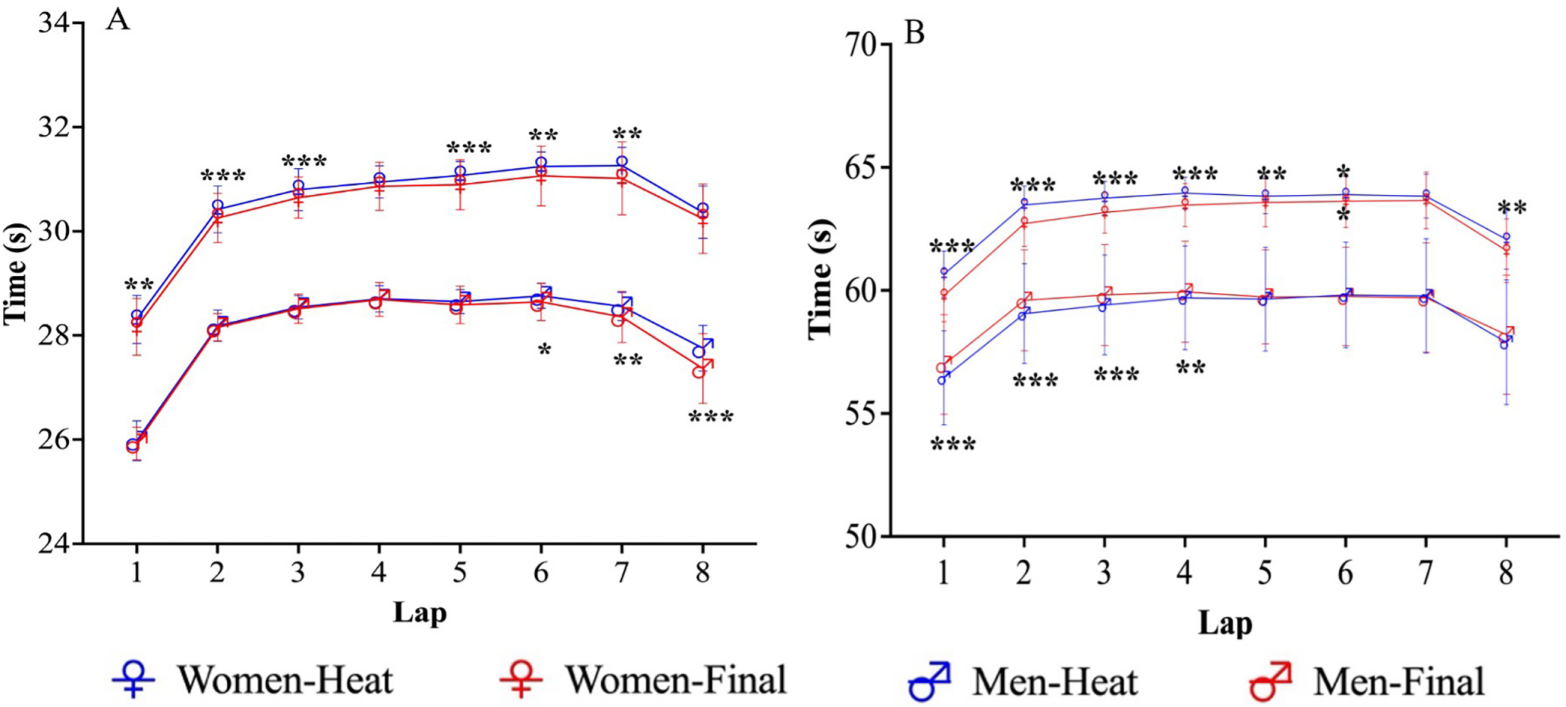
**(A,B)** Changes in different rounds of male and female freestyle swimmers (400 m freestyle event **(A)**, 800 m freestyle event **(B)**).

Figure 2 shows that in the 400 m event, the improvement in competitive performance occurred exclusively from the sixth lap and beyond (*P* < 0.05), but the female swimmers also improved significantly in the first three laps (*P* < 0.05), whereas in the 800 m event, the improvement in competitive performance all occurred in the first four laps (*P* < 0.01), and females would have a significant improvement compared to males in the second half.

### 3.2 Changes in different rounds position

**Figure 3:**
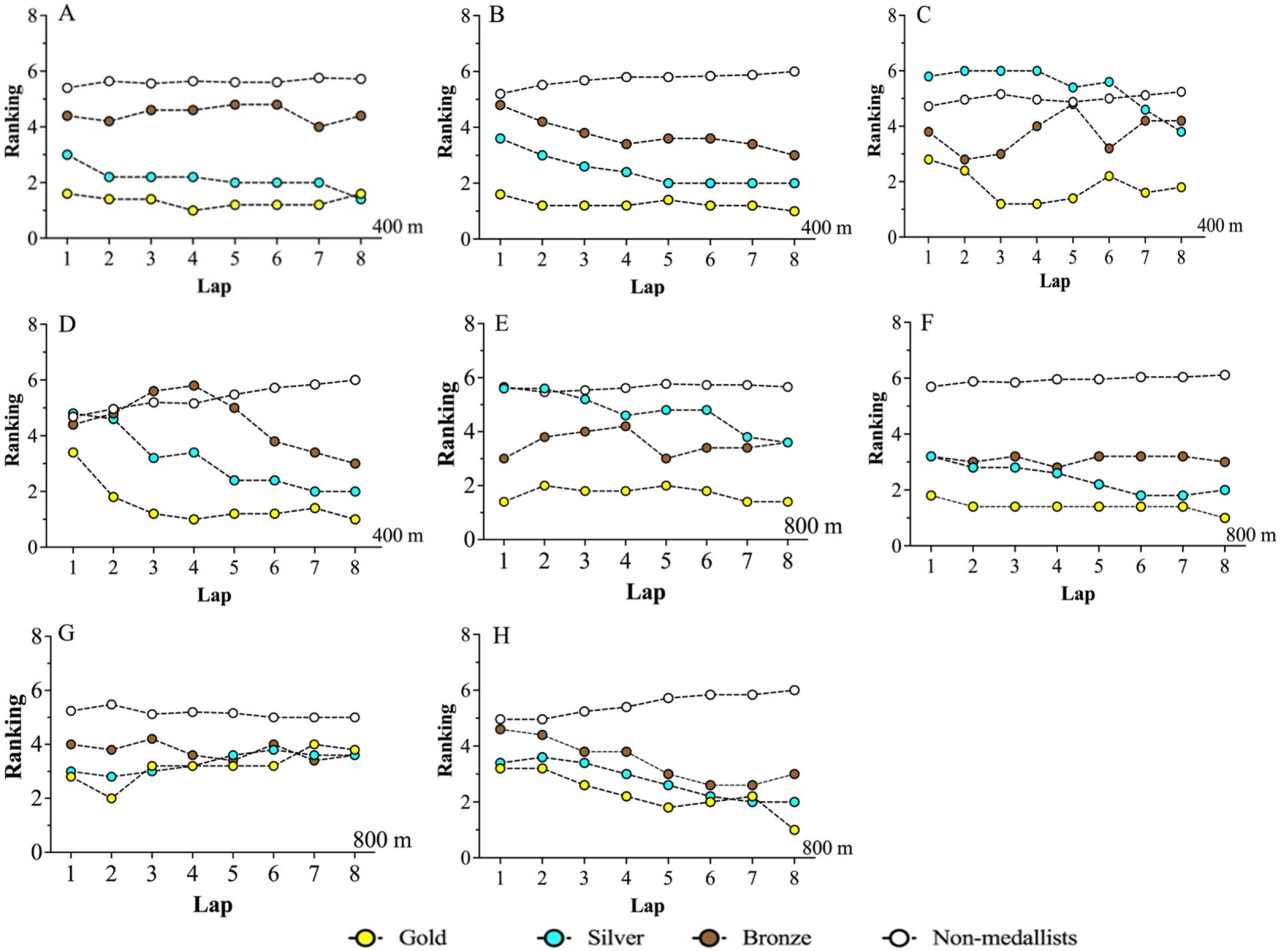
**(A–H)** Average positions of male and female swimmers in different rounds of the freestyle races (women – 400 m—heat **(A)**, women – 400 m – final **(B)**, women – 400 m - heat **(C)**, women – 400 m – final **(D)**, women – 400 m - heat **(E)**, women – 400 m – final **(F)**, women – 400 m - heat **(G)**, women – 400 m – final **(H)**).

Figure 3 shows the distribution of the average positions of all swimmers over the different rounds of the 400 m and 800 m. We suspect that gold medalists to avoid taking the first position in the heat to conserve energy. However, they tend to maintain their position relative to their own rank in the final. Specifically, in the men's 400 m heats, silver medalists generally sprinted at 300 m and overtook bronze medalists in the last 350 m, and in the final, the average positions of medalists increased significantly in the 7th lap. 5% of them were in the top three positions; whereas in the female heats, the silver medalists almost overtook their gold rivals in the last 350 m; furthermore, in the finals, the average position of the medalists increased sharply in lap 6, with the position of the gold medalist averaging no less than 1.25% and 100% in the top three positions. It is reasonable to conclude that a swimmer who is unable to increase his speed and secure a top three position by the last 100 m (lap 6) will not be able to win the meet.

In the men's 800 m heats, the gold medalists consistently reduced their position to conserve energy. In contrast, the silver medalists reduced their position to a lesser extent, while the bronze medalists’ positions fluctuated consistently in the fourth position, and in the 600 m the medalists’ positions showed a notable shift. In the final, the average position of the medalists showed a consistent upward trajectory, with the gold medalists showing a notable increase over the silver medalists during lap 7. The average position of the gold medalists remained above 2.25, with 90% of them occupying the top three positions. In contrast, the bronze medalists in the female heats showed a steady decline in position. In the final, the silver medalists began to close the gap with the bronze medalists from the 400 m onwards, and then gradually caught up with the gold medalists over the 600–700 m, but the gold medalist also initiated a sprint in the final 100 m (lap 7) to finally secure the victory, with the gold medalist's position not averaging less than 1.7 during this period, and 92.5% occupying the top three positions. Similarly, if the swimmers are not able to increase his speed in the last 100 m (lap 7) to take the lead, he will not be able to secure the championship title.

### 3.3 Changes in pacing strategy in different rounds

Despite the existence of notable discrepancies in CVs across the different rounds of both events (Table 1), they were not reflected in each lap (Figure 2), especially in the 400 m event. A two-way ANOVA was performed to examine the influence of individual differences and PS on the dependent variable. The results for the different rounds of the middle-distance freestyle events are shown in Table 3. It can be observed that the main effect of male 400 m competitor was significant in both heats and finals (*F* = 3.324, *P* = 0.007 vs. *F* = 31.199, *P* = 0.000), neither of the main effects of PS selection in different rounds was significant (*F* = 0.472, *P* = 0.628 vs. *F* = 0.004, *P* = 0.996), and neither of the interaction effects of opponent and strategy selection was significant (*F* = 0.112, *P* = 0.894 vs. *F* = 1.115, *P* = 0.327); in the 800 m, the main effect of opponent was significant in both heats and finals (*F* = 2.893, *P* = 0.098 vs. *F* = 26.156, *P* = 0.000), and the main effect of strategy choice was not significant across rounds (*F* = 0.652, *P* = 0.425 vs. *F* = 1.071, *P* = 0.38), and neither of the interaction effects of opponent and strategy choice were significant (*F* = 0.369, *P* = 0.547 vs. *F* = 0.084, *P* = 0.773).

**Table 3:** ANOVA of pacing strategy and group between different round in male and female 400 m and 800 m freestyle swimmers.

Gender	Event	Rounds	Effect	F	df	*P*
Males	400 m	Heats	Group	3.324	1	0.077
			Pacing strategies	0.472	2	0.628
			Group × Pacing strategies	0.112	2	0.894
		Finals	Group	31.199	1	0.000
			Pacing strategies	0.004	2	0.996
			Group ×Pacing strategies	1.155	2	0.327
	800 m	Heats	Group	2.893	1	0.098
			Pacing strategies	0.652	1	0.425
			Group × Pacing strategies	0.369	1	0.547
		Finals	Group	26.156	1	0.000
			Pacing strategies	1.071	1	0.308
			Group ×Pacing strategies	0.084	1	0.773
Females	400 m	Heats	Group	14,565.922	1	0.000
			Pacing strategies	9,906.561	3	0.000
			Group × Pacing strategies	9,955.16	3	0.000
		Finals	Group	14.661	1	0.001
			Pacing strategies	0.322	3	0.809
			Group ×Pacing strategies	0.794	3	0.506
	800 m	Heats	Group	9.03	1	0.005
			Pacing strategies	0.912	1	0.346
			Group × Pacing strategies	0.154	1	0.697
		Finals	Group	30.216	1	0.000
			Pacing strategies	0.024	1	0.877
			Group ×Pacing strategies	0.029	1	0.865

The female 400 m race showed a pronounced discrepancy in results between heats, with statistically significant (*F* = 14,565.922, *P* = 0.000 vs. *F* = 9,906.561, *P* = 0.000) analyses of the main effects of both competitor and choice of strategy, and notable interactions (*F* = 9,955.16, *P* = 0.000) The results of the analyses in the finals mirrored the male 400 m, and the female 800 m yielded comparable results to the male 800 m, there was a significant main effect of competitor in the heats and finals (*F* = 9.03, *P* = 0.005 vs. *F* = 30. 216, *P* = 0.000), while a non-significant main effect of strategy choice across rounds (*F* = 0.912, *P* = 0.346 vs. *F* = 0.024, *P* = 0.877) and an interaction effect of both competitor and strategy choice were also not evident (*F* = 0.154, *P* = 0.697 vs. *F* = 0.029, *P* = 0.865).

**Figure 4:**
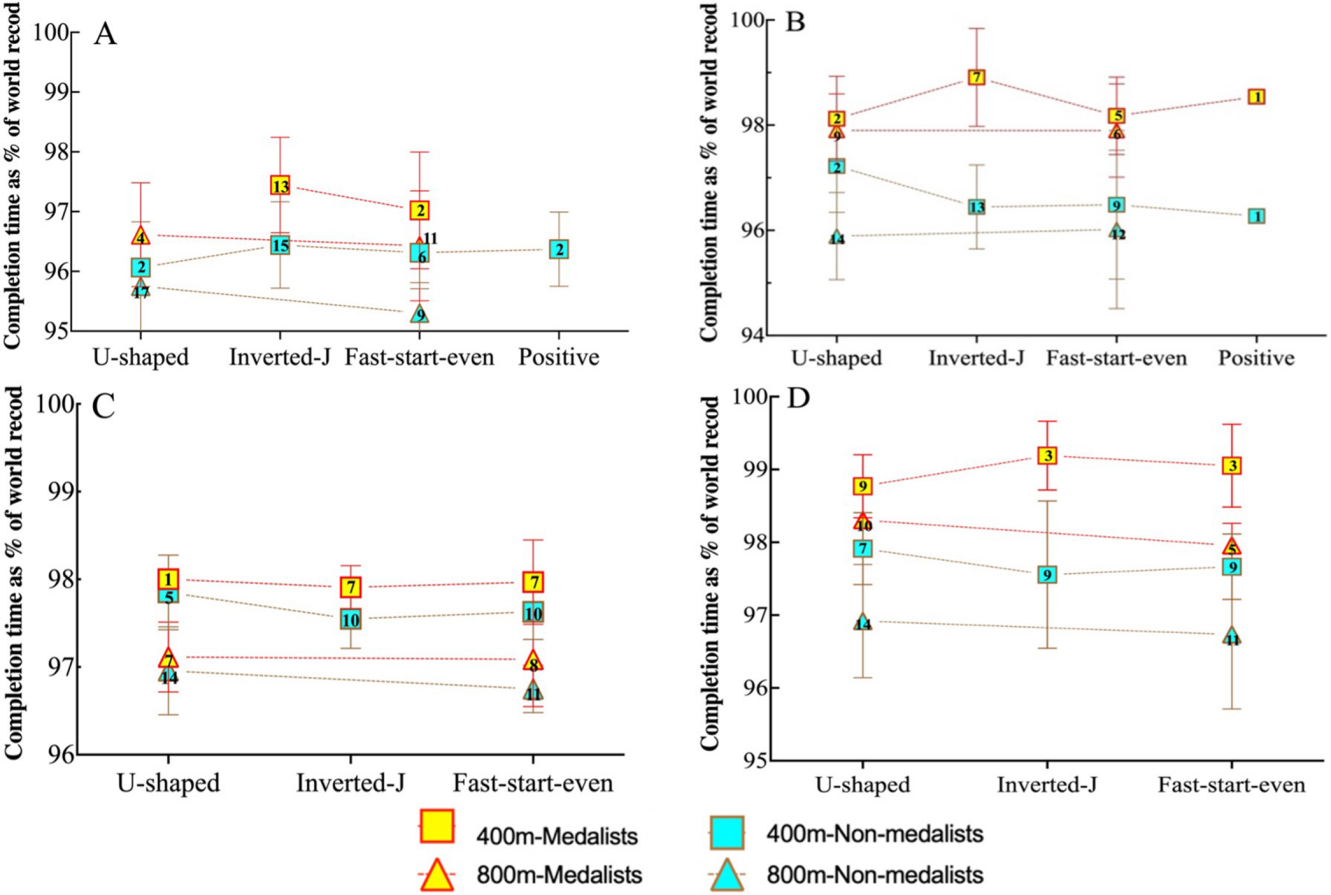
**(A–D)** Changes in pacing strategy of male and female swimmer in different rounds of the 400 m and 800 m freestyle races at the swimming world championships from 2017 to 2024 (women - heats **(A)**, women - finals **(B)**, Men - heats **(C)**, Men - finals **(D)** the numbers in the graphs represent the number of times the pacing strategy was used.

Figure 4 analyses the frequency and competitive performance of different PS. It was found that in the female 400 m freestyle, the inverted-J was identified most frequently [total count (TC) 48] and the U-shaped (TC: 6) and the positive (TC: 4) were the least used, both in the heats and in the finals. In addition, some of the strategic choices were changed from the inverted-J to the fast-start-even in the finals. In the men's 400 m freestyle, the fast-start-even (TC: 17) was used most frequently and the U-shape (TC: 6) least frequently in the heats, but the opposite was true in the finals, where the U-shape (TC: 16) was used most frequently and the fast-start-even and inverted-J were used less frequently (TC:12). In the 800 m freestyle, no one chose to use the inverted-J and the U-shape was the most frequently used strategy, regardless of gender or round. It was observed that swimmers did not prefer negative and variable strategies under any circumstances.

In the female 400 m final, medalists who chose the inverted-J were the most successful, with a mean time of 98.91 ± 0.93% [CI = 98.05–99.77%] of the WR (equivalent to a mean time of approximately 3:53.88 ± 2.20 s) and used it the most. The lowest performance rating for those who chose the U-shape was 98.12 ± 0.81% of the WR, with a consistent trend between preliminaries and final performance. However, among the non-medalists, the worst performance was observed for the positive strategy in the final and the U-shape in the heats. Similarly, the medalists in the men's 400 m final chose the inverted-J for optimal performance, achieving a rating of 99.19 ± 0.47% (CI = 98.02% - 99.87%) of the WR (equivalent to an average time of approximately 3:38.29 ± 1.04s), while choosing the U-shaped for their least successful performance rating of 98.77%. In both heats and finals, non-medalists performed best when using the U-shaped strategy, while the inverted-J was associated with the worst results.

## 4 Discussion

The swimmers analyzed in this study were at the pinnacle of their sport on a global scale, and to the best of the authors’ knowledge, this is the first time that a full round study of middle-distance freestyle swimmers has been conducted in consecutive years. The first research objective of this study was to examine the CVs and Δ between swimmers in different rounds over five World Championships. As a matter of practical speculation, a swimmer's competitive performance in subsequent rounds can be discerned from their heat results (19). The results of our study showed that 87.5% of the swimmers in the 400 m and 800 m finals had an average CVs greater than 0.5% relative to their heats, and when gender was taken into account, the change in CVs was more pronounced in female performance than in male performance, consistent with the findings of Nikkolaids & Knechtle (2017) (29). Taken together, at least 73.8% of the swimmers who had the potential to reach the finals and 26.2% of the swimmers who were failures may have been hindered by a lack of effective goal orientation or an inability to automatically execute performance under the pressure of international competition, and therefore were unable to achieve peak performance (30, 31).

The available evidence suggests that longer distance swimmers exhibit greater increases in CVs as the distance swum increases and the competition progresses to the final (23). The results of our study support this conclusion, with greater changes in the 800 m race compared to the 400 m race. In addition, the CVs improvement for medalists would be greater than for all participants, i.e., their improvement would increase to 1.07% and 1.43% for 400 m and 800 m event, respectively. This indicates that the competitive improvement of medalists was offset by the regression of non-medalists, with medalists showing greater improvement. The reason for this may be that the role of PS is not significant in non-medalist swimmers (32).And these results exceed those reported by Trewin et al. (2004) (33), who concluded that medalists improved by 0.9%. In contrast, our results are relatively aligned with Pyne et al. (2004) (2) and Cuenca-Fernandez et al. (2021) (19), they found that medalists would improve their competitive performance by up to 1.2%. This may therefore be a common characteristic of finalists. The 400 m and 800 m freestyle swimmers should all strive to improve their competitive performance by at least 1% from heats to finals in order to medal (2). In addition this, each round consisted of start, net stroke, turn, and sprint times, with turns accounting for approximately 25% of the total time, so a 2% improvement in turn time would only improve overall performance by 0.5% (34). However, the influence of changes on CVs may not be sequential, so further investigation is needed to determine whether specific component times exhibit greater variability.

The performance of swimmers is influenced by many factors including physiological, energetic, biomechanical and anthropometric characteristics (35). It is reasonable to assume that elite swimmers who fail to achieve a 0.5% improvement in CVs per lap in a major international race will not be able to fully improve their energy efficiency, thereby reducing their chances of winning medals (2). Other a study have shown that elite swimmers use conservative strategies in the 400 m and 800 m heats to improve their performance in the finals; this approach has been shown to result in higher CVs and % Δ between laps (23). In the present study, the improvement in each round from the preliminaries to the final was greater than 0.5%, particularly in the 400 m event, where the improvement in the front and back strokes was most significant. Conversely, in the 800 m, only the change in the front stroke reached statistical significance (Table 2). This finding supports the assertion that the swimmers who were able to swim at a higher speed in the finals compared to their heats. The differences in CVs between the 400 m and 800 m events in this study were minimal. Considering that each swimmer may have used multiple PSs to reach the final for the ultimate victory (5) (McGibbon et al., 2018), there may have been differences between the genders in the different laps (Figure 2). Specifically, in the 400 m event, it can be observed that females tend to be more aggressive in the beginning of the final to secure a more advanced position, while males may try to increase their speed in the last lap. This result is the same as the study by Robertson (2009) et al. who analysed each lap of nine 400 m freestyle international events and concluded that a fast first lap from the dive start is followed by mainly even pacing through the middle laps, and an evenly paced or slightly faster final lap (32). It is worth noting that in addition to the start and sprint phases, all swimmers will experience a significant acceleration from 200 m to 250 m. This pre-emptive advantage will put psychological pressure on the other swimmers and may disrupt the PS of the others s (36). Whereas in the 800 m event, both female and male swimmers exhibited a similar level of aggression at the beginning, and females showed an increased concern about their position in the back lap.

The second research objective of this study was to determine if the changes in swimmer positioning are consistent with the above. Although the swimmers involved in the finals did not compete at the same time and in the same pool, an attempt was made to visualize the positioning of all swimmers in different rounds (Figure 3). In the heats, gold medalists often conserved their energy with the apparent goal of only qualifying for the finals, especially in the female 400 m and men's 800 m. In these events, the average position of the gold medalists was further back, while the silver medalists performed better, indicating that they expended more energy. In the final, the medalists generally increased their speed from the start of the race to secure advantageous positions, the average position of the gold medalists was no lower than the top 2, and in over 90% of the eight segments they occupied the top 3 positions. Consequently, it was difficult for swimmers in the 400 m and 800 m to secure a medal unless they were in the top 3 in the final 100 m. This finding is consistent with the findings of Mytton et al. (2015) (37), who believe that the best performance of swimmers involves a combination of relatively slow initial laps and fast final laps. This suggests that most swimmers may use multiple similar PSs. It is possible that visual feedback may play an important role in this performance (38). To illustrate, in the finals, if swimmers realize that they are not among the medalists after observing the position of their opponents during the competition, they may reduce their speed in the last laps of the race. Conversely, to qualify for the finals, it is essential that the swimmers who have placed well in the other heats of the race do so, so they may decide to make an extra effort in the last laps of the race.

A previous study examining 1,500 m positioning at the World Championships found that many champions were in the top 3 throughout the final and could not be crowned champions without maintaining a top 3 position for the last 40% of the distance (17). A similar conclusion is reached in our study of shorter distances. A study of the London Olympics showed that gold medalists not only started in a favorable position but also gradually gained with the psychological advantage of “we take the initiative,” while silver and bronze medalists tended to follow each other, and non-medalists gradually dropped in the rankings (39). Therefore, it can be concluded that these position changes represent the main competitive characteristics of middle-distance swimmers.

The third research objective of this study was to gain insight into the importance of PS and its relationship to competitive performance as outlined above. The results of our study indicated that the selected PS did not significantly influence the final performance of all swimmers, except for the female 400 m heats (*P* < 0.1). In addition, the tactics used by medal group swimmers were found to be similar (*P* > 0.1), consistent with previous research (27). The discrepancy in performance between medal and non-medal swimmers was greater when the inverted-J was chosen (Figure 4), which also explains the observation that only female swimmers appeared significant in the second half of the rounds, while the first half of the rounds did not coincide with the male (Figure 2).

The use of positive strategies is observed to be minimal, with only one female swimmer using such a strategy. Furthermore, this strategy is conspicuously absent from the discourse surrounding other 400 m and 800 m freestyle studies. However, there are exceptions to this, as evidenced by the findings of Tucker et al. (2006) (40), who discovered that the use of a positive strategy enabled the establishment of 26 WR in the 800 m freestyle. Therefore, it can be hypothesized that the use of a positive strategy is an inefficient approach in middle-distance swimming, which would explain the relatively low selection rate of elite swimmers. In addition, in the 400 m heats, female swimmers were more likely to use the inverted-J and males the fast-start-even, and in the finals, females continued to use the inverted-J (98.91% WR), and males shifted to the U-shaped (98.77% WR), which is consistent with the view of the previous study (15). In the 800 m, both male and female swimmers uniformly used the U-shape (99.92% WR and 97.90% WR, respectively). This seems to indicate that these strategies are a contributing factor to the best performance in middle distance swimming, with these PS resulting in times that are very close to the most recent WR. Of course, these pacing strategies may be empirically oriented among competitors or may be conscious choices (27), making them difficult to replicate. However, some swimmers may benefit from them (41, 42). An interesting observation is that the U-shape in heats and the inverted-J in finals (chosen by a limited number of swimmers) produced the shortest times in the men's 400 m (98.0% WR and 99.19% WR, respectively). Possible explanations are (1) the fastest swimmers chose to adapt only to this strategy, (2) these strategies generate some kind of tactical advantage (e.g., positional, psychological, etc.) in different rounds, and (3) it may depend on other competitors. Despite the existence of this type of PS in a study conducted a decade ago (27), strangely enough, negative and variable strategies are rarely used nowadays. This may be because, with increased technological support, swimmers have changed their choice of energy distribution and cognition of pacing strategies for science in the middle-distance swimming race.

The PS may be an intrinsic reflection result or an organismic regulatory mechanism of the swimmer. To illustrate, a study of 400 m swimming using a breath analyzer revealed that oxygen uptake levels were associated with the ability to maintain a high swolf (professional index of swimming efficiency). The results indicated that the higher swolf of the swimmers was associated with delayed fatigue and the conservation of anaerobic energy supply during the last laps, where the stroke length (SL) decreased from lap to lap and the SR was U-shaped (15). Therefore, it can be concluded that most male swimmers use a fast-start-even or U-shaped strategy.

In addition, a fast-start-even strategy has been observed to increase oxygen consumption more rapidly and more markedly, allowing for the maintenance of a superior average speed on the back lap (6). 800 m medalists showed a greater divergence in all speed indices from non-medalists in the final laps, suggesting that they were able to draw on energy reserves that non-medalists were unable to mobilize (37). According to Burnley & Jones, (2007) (43), medalists using the U-shaped strategy have lower physiological barriers in the final laps, probably because they have faster oxygen kinetics and higher critical velocity, and on the other hand, they require a longer time to reach VO2max, while reducing unfavourable physiological elements and exercise-induced fatigue during intermediate decelerations. This process preserves the anaerobic reserve and subsequently calls upon the remaining anaerobic reserve to sprint at a higher speed.

It should be emphasized that the study of changes in competitive performance and power over rounds is crucial for elite swimmers. However, most studies in this area have focused on retrospective analyses of race data, with factors such as physiological biochemistry, biomechanics, and psychology in real races influencing these changes. Even coaching perceptions (44) and the environment (45) can have an impact. Therefore, the observed effects and mechanisms remain speculative. Currently, the World Aquatics has a new friendly rule change in swimwear and wearables (AQUATICS) (46), which suggests the installation of a camera in future studies and the use of micro wearable devices to assist in monitoring the SR and SL of swimmers in different rounds. In addition, it allows the measurement of physiological and biochemical components before and after the race to deepen the practicality of our application of this paper.

There is a notable lack of research on how swimmers learn and develop PS (47). Previous research has shown that swimmers competing in the 400 m freestyle are also able to perform better in the 200 m, 800 m, and 1,500 m due to the presence of an analogous energy supply system (48). In conjunction with our findings that benign migration of PS is possible between the 400 m and 800 m, and the reality that no swimmer has ever won gold medals from 50 m to 1,500 m freestyle simultaneously, the field of variability in competitive performance and PS from different laps in all freestyle events could be investigated in the future. It would be beneficial for swimming coaches from evidence-based guidelines in this area to implement individualized training in race tactics (15) and swimmers could be adequately prepared for competition (16).

## 5 Conclusion

In summary, approximately three-quarters of the participants showed an improvement in performance in the finals, with medalists improving more and non-medalists regressing slightly. Positioning choices vary depending on the swimmer's event, round, and capacity. For example, in preliminaries, a common strategy used by gold medalists is to conserve energy, with silver medalists performing better, while in finals, a common strategy used by medalists is to increase their speed from the beginning of the race to secure a favourable position. In addition, it is extremely difficult to secure a medal if a swimmer is unable to finish in the top 3 in the last 100 m of the 400 m and 800 m events. The choice of the PS that most closely matches the WR can be influenced by the above factors as well as the swimmer's gender. For example, in the 400 m heats, female swimmers were more likely to use the inverted-J, fast-start-even for males, and in the finals, females continued to use the inverted-J while males shifted to the U-shaped. In the 800 m, all swimmers uniformly used the U-shape. This study highlights the variability in competitive performance and PS across rounds, providing strategy decisions for elite swimmers and coaches.

## Data Availability

The original contributions presented in the study are included in the article/Supplementary Material, further inquiries can be directed to the corresponding author.
